# Removal of bacterial and viral indicator organisms in full-scale aerobic granular sludge and conventional activated sludge systems

**DOI:** 10.1016/j.wroa.2019.100040

**Published:** 2019-12-26

**Authors:** Mary Luz Barrios-Hernández, Mario Pronk, Hector Garcia, Arne Boersma, Damir Brdjanovic, Mark C.M. van Loosdrecht, Christine M. Hooijmans

**Affiliations:** a Department of Environmental Engineering and Water Technology, IHE-Delft Institute for Water Education, P.O. Box 3015, 2601, DA, Delft, the Netherlands; b Department of Biotechnology, Delft University of Technology, Van der Maasweg 9, 2629, HZ, Delft, the Netherlands; c Royal HaskoningDHV B.V., P.O Box 1132, 3800, BC, Amersfoort, the Netherlands

**Keywords:** Aerobic granular sludge, Activated sludge, Faecal indicators, F-specific RNA bacteriophages, Pathogen removal

## Abstract

The aim of this study was to evaluate the effectiveness of the novel aerobic granular sludge (AGS) wastewater treatment technology in removing faecal indicator organisms (FIOs) compared to the conventional activated sludge (CAS) treatment system. The work was carried out at two full-scale wastewater treatment plants (WWTP) in the Netherlands, Vroomshoop and Garmerwolde. Both treatment plants have a CAS and AGS system operated in parallel. The parallel treatment lines are provided with the same influent wastewater. The concentrations of the measured FIOs in the influent of the two WWTPs were comparable with reported literature values as follows: F-specific RNA bacteriophages at 10^6^ PFU/100 mL, and *Escherichia coli* (*E. coli*), *Enterococci*, and Thermotolerant coliforms (TtC) at 10^5^ to 10^6^ CFU/100 mL. Although both systems (CAS and AGS) are different in terms of design, operation, and microbial community, both systems showed similar FIOs removal efficiency. At the Vroomshoop WWTP, Log_10_ removals for F-specific RNA bacteriophages of 1.4 ± 0.5 and 1.3 ± 0.6 were obtained for the AGS and CAS systems, while at the Garmerwolde WWTP, Log_10_ removals for F-specific RNA bacteriophages of 1.9 ± 0.7 and 2.1 ± 0.7 were found for the AGS and CAS systems. Correspondingly, *E. coli*, *Enterococci*, and TtC Log_10_ removals of 1.7 ± 0.7 and 1.1 ± 0.7 were achieved for the AGS and CAS systems at Vroomshoop WWTP. For Garmerwolde WWTP Log_10_ removals of 2.3 ± 0.8 and 1.9 ± 0.7 for the AGS and CAS systems were found, respectively. The measured difference in removal rates between the plants was not significant. Physicochemical water quality parameters, such as the concentrations of organic matter, nutrients, and total suspended solids (TSS) were also determined. Overall, it was not possible to establish a direct correlation between the physicochemical parameters and the removal of FIOs for any of the treatment systems (CAS and AGS). Only the removal of TSS could be positively correlated to the *E. coli* removal for the AGS technology at the evaluated WWTPs.

## Highlights

• No difference in removal efficiencies (α = 5%) of FIOs was found for the studied AGS and CAS systems.
• 1.3 to 2.1 Log_10_ removals for F-specific RNA bacteriophages were found for the CAS and AGS systems.
• 1.1 to 2.3 Log_10_ removals for *E. coli*, *Enterococci*, and TtC were obtained for the AGS and CAS systems.
• The two stage CAS performed better than the carrousel CAS in removing F-specific RNA bacteriophages.
• A positive correlation was found between the TSS and *E. coli* removal, and COD and TtC removal in the AGS systems.

## Graphical abstract

**Figure:**
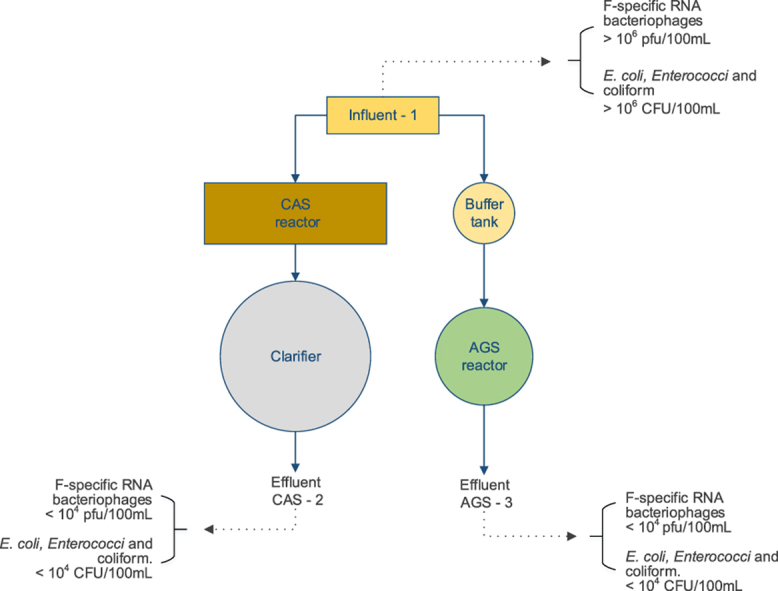

## 1 Introduction

Pathogens enter the aquatic environment through municipal wastewater discharges; their occurrence in either treated or raw wastewater may contribute to spreading epidemiological waterborne diseases (Bijkerk et al., 2015; Efstratiou et al., 2017). Several studies have reported that conventional wastewater treatment plants (WWTP) do not completely remove pathogens (Dias et al., 2018; Lucena et al., 2004; Ma et al., 2016; Ottoson et al., 2006a). For instance, Lodder and de Roda Husman (2005) and van Beek et al. (2016) reported the presence of noroviruses and enteric viruses in river basins in the Netherlands originating from treated municipal wastewater discharges.

Microorganisms such as *E. coli* (gram-negative), *Enterococci* (gram-positive) and total coliforms are commonly used as indicators for faecal contamination (Carré et al., 2018; Liu et al., 2018), and as water quality standards for water reuse (E.U, 2016). Bacteriophages have been used as an indicator for the occurrence of viral pathogens (Amarasiri et al., 2017; Dias et al., 2018; McMinn et al., 2017); particularly, the male-specific phages (F-specific RNA bacteriophages) have been used as indicators considering their strong survival in wastewater treatment process and other water environments (Mandilara et al., 2006). Furthermore, F-specific RNA bacteriophages characteristics, such as their isoelectric point, size (22–29 nm), and morphology (Grabow, 2001; Lodder and de Roda Husman, 2005) resemble human viruses such as noroviruses and other enteric viruses (Fauvel et al., 2017). Bacteriophages are also easier to detect than human viruses; inexpensive conventional analytical methods can be used to determine them.

The municipal wastewater treatment worldwide, and particularly in The Netherlands, where this research was carried out, aims at removing organic matter and nutrients (Roeleveld et al., 2010). The conventional activated sludge (CAS) systems is one of the most commonly applied technologies. This technology removes carbon (C), phosphorus (P) and nitrogen (N) by treating the raw wastewater in different compartments (biological reactors); the treated wastewater is finally separated from the biomass by a secondary clarification tank and discharged into the receiving water body (Orhon, 2015; Roeleveld et al., 2010). CAS systems can achieve over 90% removal of C, P, and N. Moreover, CAS systems are effective in removing pathogens; Matthews et al. (2010) and Amarasiri et al. (2017) reported removal efficiencies of faecal indicator organisms (FIOs) such as F-specific RNA bacteriophages and total coliforms of 99.5% and higher. FIOs may be either physically removed, being enmeshed into the flocs during the sedimentation process (Chahal et al., 2016; Fauvel et al., 2017; Guzman et al., 2007), or biologically predated by other high order organisms such as protozoa (Mallory et al., 1983).

Aerobic granular sludge (AGS) wastewater treatment systems have recently emerged as an alternative to the CAS process. AGS systems simultaneously remove organic matter and nutrients in a single compartment by promoting the formation of a granular biofilm (Beun et al., 1999). The bacteria are able to aggregate, forming dense granules of a spherical shape with a much higher settling velocity compared to CAS sludge flocs; therefore, the separation of the biomass (granules) from the treated water can be easily achieved by sedimentation (de Kreuk et al., 2007; Khan et al., 2013). Both the biological degradation of organic matter and nutrients, and the solid-liquid separation process occurs in a single basin. According to Pronk et al. (2015), full-scale AGS systems may reach C, P, and N removals on average of 87, 86, and 86%, respectively. However, the performance of full-scale AGS systems on pathogen removal is still unknown.

The objective of this study was to compare the removal of FIOs in two full-scale wastewater treatment plants in the Netherlands provided with both AGS and CAS systems operated in parallel. The AGS and CAS systems were evaluated on their removal of F-specific bacteriophages, *E. coli*, *Enterococci* and thermotolerant coliforms (TtC). Standard water quality parameters such as chemical organic matter (COD), ammonium (NH_4_–N), biological organic matter (BOD_5_), orthophosphate (PO_4_–P), total suspended solids (TSS), and their relation with the removal of the microbiological organisms, was evaluated as well, in order to see whether water quality parameters can be used to predict FIOs removals.

## 2 Materials and methods

### 2.1 Treatment facilities

**Fig. 1:**
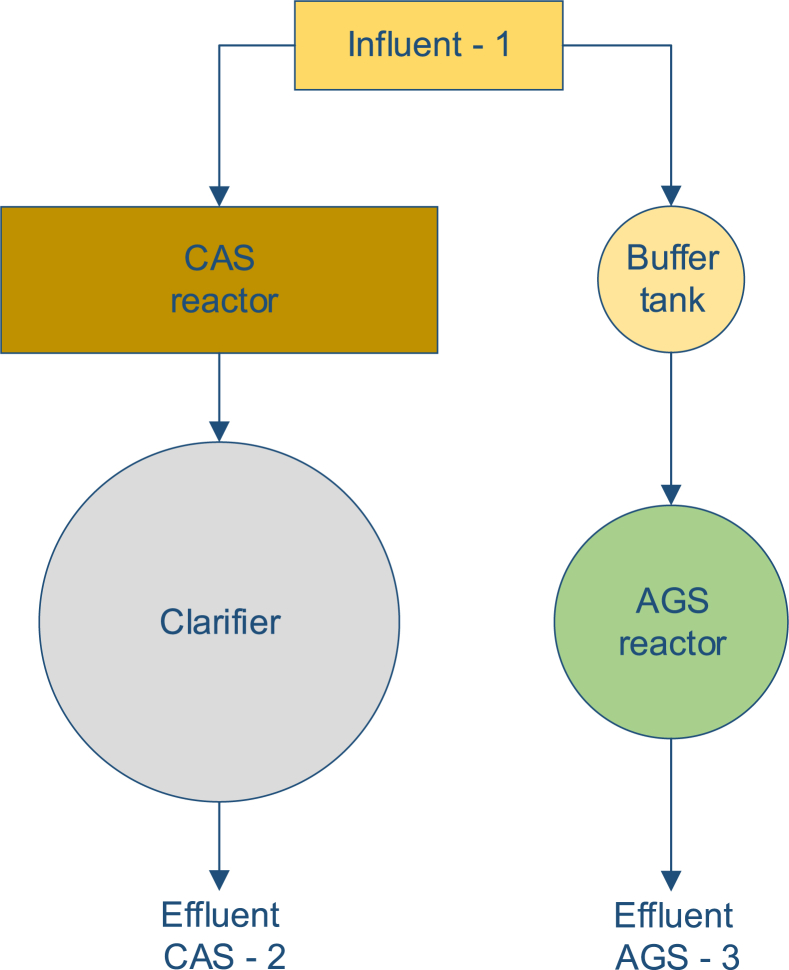
Scheme of the combined wastewater treatment plants at Vroomshoop and Garmerwolde WWTPs.

This research was performed at two different wastewater treatment plants (WWTP) in The Netherlands, Vroomshoop (52°26′49.0″N 6°33′52.6″E) and Garmerwolde (53°14′51.8″N 6°40′19.6″E); (Pronk et al., 2015). The WWTPs were initially designed as CAS systems for treating municipal wastewater; later on, AGS systems were incorporated in both WWTPs. Both processes at the two WWTPs (CAS and AGS) work in parallel receiving the same wastewater; moreover, both of them are lacking primary and tertiary treatment (Fig. 1).

The CAS systems operate in a continuous flow mode, and were designed to remove organic matter (C) and nutrients (N and P). The Vroomshoop WWTP was designed as a biological carrousel CAS system, while the Garmerwolde WWTP as a two stage AB (adsorption/bio-oxidation) CAS configuration with ferric iron dosing in the unit A for P removal. The AGS systems located at both WWTPs consist of a buffer tank (designed to store the influent raw wastewater), followed by a biological tank, containing the granular biomass where all conversions simultaneously occur. The AGS systems are operated as sequencing batch reactors. Table 1 describes the operational conditions for each treatment system at each WWTP.

**Table 1:** Operational conditions of the two treatment systems (CAS and AGS) at the Vroomshoop and Garmerwolde WWTPs during the sampling campaign - December 2017 to May 2018.

Parameters	Vroomshoop		Garmerwolde	
	CAS	AGS	CAS	AGS
Average Dry flow (ADF) (m^3^/d)	2140	1541	27,645	20,355
Average peak flow (m^3^/h)	1125	140	7148	4200
Population equivalent COD based	13,100	9500	210,000	140,000
Organic loading rate (kg COD/m^3^/d) (aerated)	0.50	0.65	0.53	0.50
Mixed liquor suspended solids range (g/m^3^)	3–6	9–14	5–8	10–14
Aerated basin, O_2_ (g/m^3^)	0.3–1.8	1.5–2.8	1.0–2.0	0.2–2.4
Water temperature (^◦^C) range	8.5–17.8	8.5–17.8	8.6–18.0	8.6–8.0
Hydraulic retention time (HRT) at ADF(h)	27	11–24	24	10–12
Sludge retention time (d)	27	>21	23	>30

### 2.2 Sample collection

Grab samples were collected once a month on two consecutive days from December 2017 to May 2018 for each WWTP at three different sampling points: (1) influent wastewater; (2) CAS treated effluent; and (3) AGS treated effluent. The influent wastewater samples, (shown in Fig. 1 as influent-1) represent the raw municipal wastewater after passing through a grit removal process, and before reaching either the CAS system, or the AGS system’s buffer tank. The CAS treated effluent samples (shown in Fig. 1 as effluent CAS-2) were taken directly from the overflow weir of the CAS settling tank. The AGS treated effluent samples (shown in Fig. 1 as effluent AGS-3) were collected from the effluent discharge channel after completing one entire batch cycle. The water temperature during the sample campaign ranged between 9 °C and 15 °C.

### 2.3 Sample analysis

The influent and effluent samples were stored in plastic bottles, and were shipped in dark containers refrigerated at 4 °C within 24 h to two different laboratories as follows. Bacteriophages were determined at the WNL laboratory (Glimmen, the Netherlands). Other microbiological (*E. coli*, TtC, *Enterococci*) and physicochemical parameters (COD, BOD_5_, NH_4_–N, PO_4_–P, and TSS) were determined at the ALS laboratory (Prague, Czech Republic). Water temperature and DO concentrations were collected directly from the WWTPs at the same time the samples were taken. The determinations of the physicochemical parameters were carried out according to the standard methods APHA (2012) for the examination of water and wastewater. The determination of the microbiological parameter is explained below.

#### 2.3.1 Bacteriophages detection

F-specific RNA bacteriophages were enumerated in duplicate following the double layer method according to ISO 10705-1 (Anon, 2000c). The samples were 10, 100, and 1000-folds diluted in a saline water solution, and then one (1) mL of sample was mixed together with one (1) mL of 3 h cultured bacterium host *Salmonella typhimurium* strain WG49. Each sample was mixed in a semi-solid nutrient agar, and poured in a solid nutrient agar plate. Samples were then incubated at 37 °C for 18 h. For enumeration, each plaque in the bacterial mat was counted as one bacteriophage unit. F-specific RNA bacteriophages were determined by plaque-forming units (PFU); the detection limit was reported at 1 PFU/100 mL.

#### 2.3.2 Bacteria enumeration

The analytical determinations of *E. coli* and TtC bacteria were performed following the membrane filtration technique for enumeration according to the standard CSN 75 7835, which is a modified method of the standard method ISO 9308-1 (Anon, 2000b) for samples with excessive growth of the accompanying microflora. For enumeration, samples were 10, 100, and 1000-folds diluted in a phosphate-buffered saline (PBS) solution. Samples were plated on *Chromocult*® (Merck Millipore) medium agar for *E. coli* detection and plated in 4-methylumbelliferyl-beta-D-glucuronide *E. coli* broth for TtC detection. Later, they were cultivated 24 h at 37 ± 2 °C and 44 ± 2 °C for *E. coli* and TtC, respectively.

*Enterococci* detection and enumeration was performed by the same membrane filtration method according to the standard ISO 7899-2 (Anon, 2000a). The samples were cultivated in the Slanetz and Bartley Medium (Oxoid^TM^) for 24 h at 44 ± 2 °C. Confirmatory colour reaction tests were also performed to discard false positive by placing the samples in a Bile Esculin Agar (Sigma-Aldrich®, Germany).

All bacteria (*E. coli,* TtC and *Enterococci*) were enumerated by agar plate colony forming units (CFU); the detection limit was reported at 1 CFU/100 mL.

### 2.4 Data analysis

A statistical analysis was performed on the twelve samples collected at each WWTP for each target organism. Data is presented in Box-Whisker plots in which the horizontal line across each box represents the median, the interquartile ranges (50% of the score of the data) and the outliers represent the confident limit of 95% of the determined concentrations. FIOs concentrations were converted to Log_10_. The removal efficiencies (Log_10,_ %) were calculated considering the concentration of the influent wastewater reaching the WWTPs and the treated effluent discharges for each specific treatment process (AGS and CAS).

Shapiro-Wilk normality test was applied to the converted Log_10_ concentrations of the target FIOs, to the unconverted data of the studied physicochemical parameters and their corresponded removal efficiencies. The Log_10_ FIO removal showed to be normally distributed, therefore the two-paired Student’s t-distribution (t-test 95% confidence) analyses were performed to check whether statistically significant differences on the FIOs removal efficiencies exist, on (i) similar processes (that is, CAS at Vroomshoop versus CAS at Garmerwolde, and AGS at Vroomshoop versus AGS at Garmerwolde); and (ii) on different processes at each WWTP (that is CAS versus AGS at both Vroomshoop and Garmerwolde WWTPs). A *p*-value ≤ 0.05 was used to bind the statistical significance.

Moreover, Pearson’s product correlation analyses were performed to determine the possibility for establishing potential trends among the FIOs removal efficiencies; and the Spearman’s rank relationship was applied to the comparison of (i) the FIOs and physicochemical concentrations of the influent wastewater, (ii) the FIOs removal efficiency with the removal efficiency of the target standard water quality parameters. The statistical analysis was carried out to determine the presence of any association by calculating the product correlation value from −1 to 1. Moreover, the *p-*value for each correlation was calculated to determine whether the association was significant (*p*-value < = 0.05) or not. Calculations were statistical computing using R (R Core Team, 2019).

## 3 Results

### 3.1 FIOs concentrations in raw and treated wastewater

**Fig. 2:**
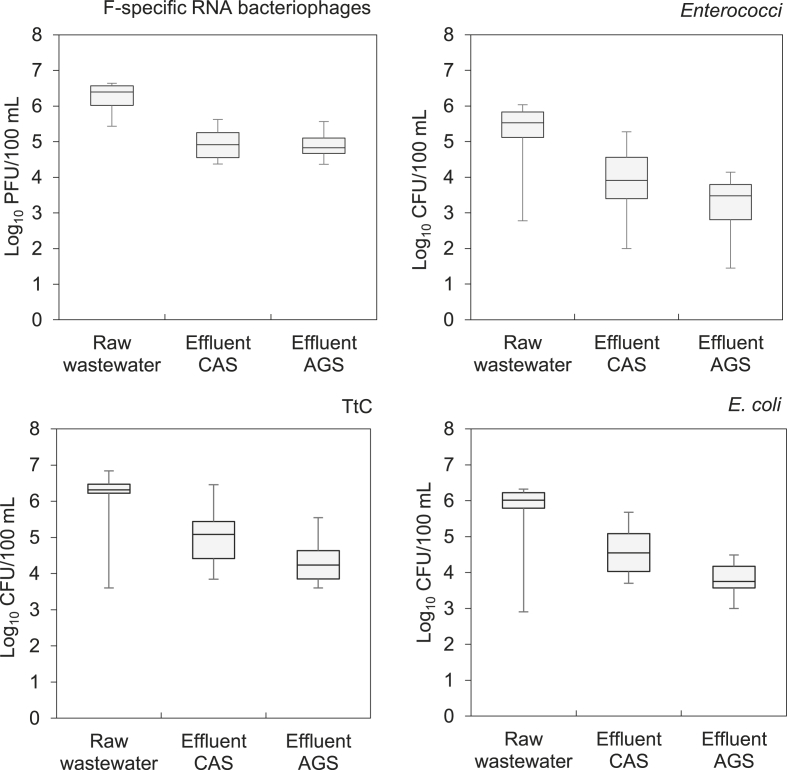
FIOs concentrations per sampling point at Vroomshoop WWTP, The Netherlands (December 2017 – May 2018); number of samples n=12.

**Fig. 3:**
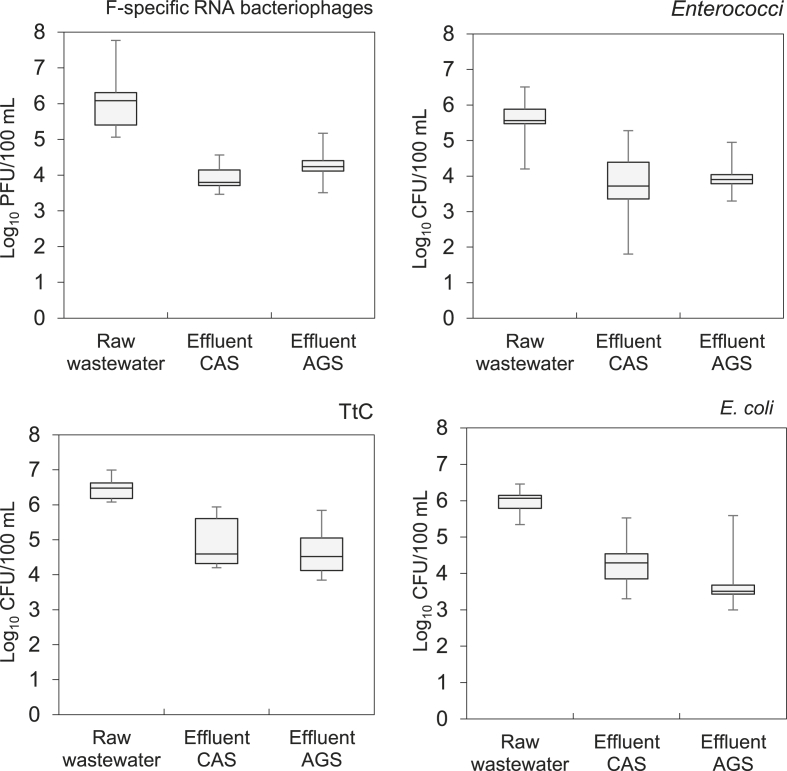
FIOs concentrations per sampling point at Garmerwolde WWTP, The Netherlands (December 2017 – May 2018), number of samples n=12.

Fig. 2 and Fig. 3 show Box-Whisker plots of the FIOs concentrations obtained from each sampling point at the Vroomshoop and Garmerwolde WWTPs, respectively. This corresponds to the microorganism concentration in the raw wastewater (influent to the plant) and the treated effluent from each treatment system (AGS and CAS).

Regarding influent flow wastewater, F-specific RNA bacteriophages were detected in both WWTPs at an arithmetic mean concentration of 10^6^ PFU/100 mL; *Enterococci* were detected at mean concentrations of 10^5^ CFU/100 mL; and TtC and *E. coli* bacteria both at the same order of magnitude (10^6^ CFU/100 mL) in both WWTPs. The average concentrations for each of the evaluated FIOs were of similar magnitude for the two WWTPs. However, no significant correlation was found between the occurrences of the four groups of organisms (*p*-value > 0.05). The main variations in the FIOs concentrations observed during the sampling campaign (minimum and maximum values shown in Fig. 2, Fig. 3) can be explained by the hydraulic and seasonal variations when conducting this evaluation. The daily influent flow rate of both WWTPs fluctuates considerably between rain and dry weather flow. At the Vroomshoop WWTP, the minimum influent concentrations for all the target organisms were approximately between one to three orders of magnitude lower than the average concentrations. These lower values were measured in samples that were diluted with rain water because they were taken during rainy weather flow (RWF) events, with a flowrate of around 800 m^3^/h being more than 63% of the operational design average peak flow (Table 1). Moreover, variations from one to four orders of magnitude were observed at the Garmerwolde WWTP. High values were measured in samples taken at a dry weather flow period with a reported flowrate of 2300 m^3^/h, corresponding to only 15% of the operational design average dry flow at Garmerwolde WWTP. Although fluctuations in the influent wastewater flow were observed, which had an effect on the concentrations of the target organism, the correlation was not significant. Moreover, also no significant correlations were found between the water temperature (that gradually increased from 9 °C in winter to 15 °C in spring), and the concentrations of the target organisms.

The mean concentrations of the target organisms in the treated effluent of the different processes (AGS and CAS) were not significantly different for the two studied WWTPs. For Vroomshoop the average concentrations of the different FIOs in the AGS treated effluent were slightly lower than the CAS treated effluent. However, the minimum and maximum concentrations showed a high variation. For Garmerwolde, the average concentration of the different FIOs were of the same order of magnitude in the AGS and CAS treated effluent. Minimum and maximum followed similar tendency as at Vroomshoop.

### 3.2 Log_10_ removal

**Fig. 4:**
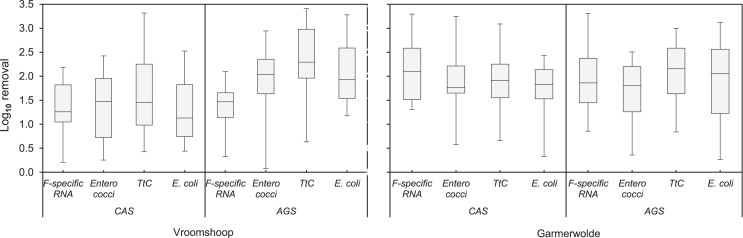
Log_10_ removal per treatment plant for AGS (left side) and CAS (right side) systems.

The average Log_10_ removals and the standard deviations for the different evaluated FIOs per treatment plant are shown in Fig. 4. The *p*-values obtained from the statistical analysis are presented in Table 2. Log_10_ removal values for F-specific RNA bacteriophages of 1.34 ± 0.60 (95.5% removal) and 2.13 ± 0.69 (99.3% removal) were reported for the CAS carrousel configuration at Vroomshoop and for the AB-CAS configuration at Garmerwolde, respectively. This difference is statistically significant (*p*-value = 0.007; <0.05). No significant statistical differences (*p-*value > 0.05) could be reported for the rest of the evaluated FIOs between the different CAS systems. *E. coli* average Log_10_ removals of 1.12 ± 0.69 (94.4% removal) and 1.65 ± 0.68 (97.8% removal) were reported at the Vroomshoop and Garmerwolde CAS WWTPs, respectively. Correspondently, values of 1.60 ± 0.85 (97.3% removal) and 1.88 ± 0.80 (98.7% removal) were reported for TtC at Vroomshoop and Garmerwolde CAS WWTPs, respectively; and 1.33 ± 0.69 (95.5% removal) and 1.92 ± 0.65 (98.8% removal) for *Enterococci* at Vroomshoop and Garmerwolde CAS WWTPs, respectively.

**Table 2:** *p*-values from t-test (95% confidence) of the comparison between similar wastewater treatment processes, and *p*-values and R-values from the comparison between different the processes at the each WWTP.

Microorganism	Similar processes comparison		WWTP comparison	
	**CAS vs. CAS**	**AGS vs. AGS**	**Vroomshoop** **CAS-AGS**	**Garmerwolde** **CAS-AGS**
F-specific RNA bact.	0.007	0.069	0.868	0.405
*Enterococci*	0.073	0.336	0.069	0.372
TtC	0.623	0.284	0.077	0.585
*E. coli*	0.239	0.383	0.086	0.543

Regarding the AGS treatment systems, no significant statistical differences (*p*–value > 0.05) were reported when comparing the Log_10_ removal of the evaluated FIOs at the AGS systems located at the two evaluated WWTPs (Vroomshoop versus Garmerwolde). F-specific RNA bacteriophages Log_10_ removals of 1.38 ± 0.50 (95.8%) and 1.88 ± 0.74 (98.6%), *E. coli* Log_10_ removals of 1.29 ± 0.64 (94.9%) and 1.86 ± 0.94 (98.6%), TtC Log_10_ removals of 2.26 ± 0.78 (99.5%) and 2.05 ± 0.69 (99.0%), and *Enterococci* 1.96 ± 0.47 (98.9%) and 1.67 ± 0.66 (97.9%) were measured for the Vroomshoop and Garmerwolde WWTPs, respectively.

Comparing the two different wastewater treatment processes (AGS versus CAS), no significant statistical differences (*p*-value > 0.05) were observed for the removal of the FIOs by the two treatment technologies at the two evaluated WWTPs.

### 3.3 Correlation between microbial organisms and water quality related parameters

Except for TSS at Vroomshoop, F-specific RNA bacteriophage concentrations in the influent wastewater at both WWTPs significantly correlated with the concentrations of the measured physicochemical water quality parameters. The rest of the bacteria indicators showed better correlations with the physicochemical parameters at Garmerwolde WWTP compared to Vroomshoop. Regarding the FIOs removal efficiency, the F- specific RNA bacteriophage removal measured at Garmerwolde WWTP significantly correlated with TtC removal (*p* =0.03) in the CAS system, and with *Enterococci* removal (*p* = 0.0004) in AGS system, but no significant correlations were found at Vroomshoop WWTP. Additional correlation products and *p*-values can be found in the Supplementary Material Tables S1 and S2.

**Fig. 5:**
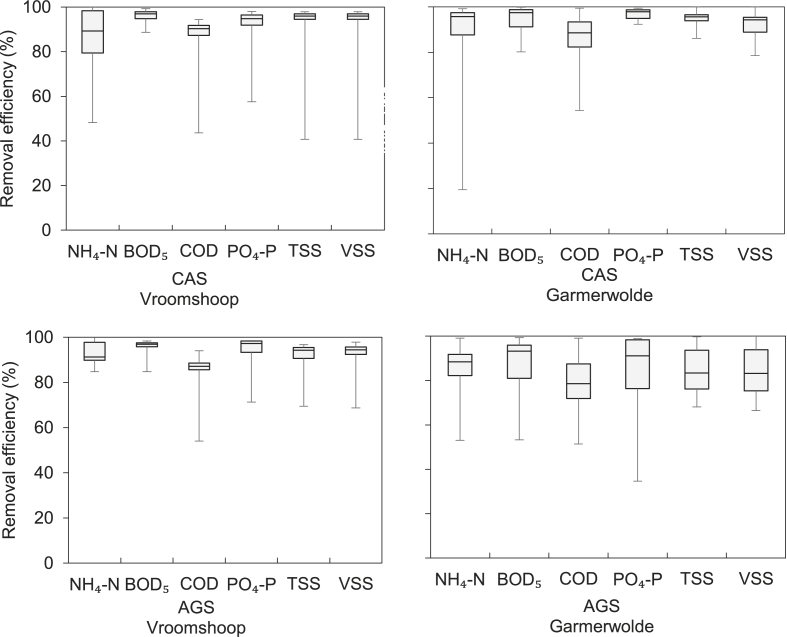
Physicochemical water quality parameters removal efficiency (%) for the two different process (CAS and AGS) at each WWTP.

Fig. 5 shows the water quality parameters (NH_4_–N, BOD_5_, COD, PO_4_–P and TSS) removal efficiency (%) for the two different process (CAS and AGS) at each WWTP. No significant difference between the WWTPs and processes was measured. The above reported FIOs removal was compared with the measured physicochemical water quality parameters per process (CAS or AGS) in each WWTP, in order to evaluate the potential correlation. Table 3 presents the Spearman’s rank coefficient (*rho*) and its corresponding *p-*values. The emphasised values (bold) correspond to statistically significant correlations (*p* < 0.05).

**Table 3:** Spearmen rank correlation obtained by correlating the removal of FIOs and the physicochemical parameters per WWTP.

		CAS							
		**Vroomshoop**				Garmerwolde			
		**F-specific RNA**	***Enterococci***	**TtC**	***E. coli***	**F-specific RNA**	***Enterococci***	**TtC**	***E. coli***
NH_4_–N	*rho*_CAS_	0.678	0.741	0.497	0.608	0.062	0.440	0.727	0.161
	*p*_-value_	**0.019**	**0.008**	0.104	**0.040**	0.851	0.154	**0.010**	0.619
BOD_5_	*rho*_CAS_	0.478	0.384	0.336	0.413	0.559	−0.427	0.210	0.126
	*p*_-value_	0.115	0.218	0.286	0.185	0.062	0.169	0.514	0.700
COD	*rho*_CAS_	0.009	0.245	0.140	0.181	0.552	−0.441	0.175	0.214
	*p*_-value_	**0.030**	0.444	0.670	0.573	0.067	0.154	0.588	0.499
PO_4_–P	*rho*_CAS_	0.713	0.657	0.420	0.755	0.664	−0.448	0.056	0.203
	*p*_-value_	**0.012**	**0.024**	0.177	**0.007**	**0.020**	0.147	0.869	0.528
TSS	*rho*_CAS_	0.734	0.720	0.503	0.671	0.671	−0.224	0.231	0.385
	*p*_-value_	**0.009**	**0.011**	0.099	**0.020**	**0.020**	0.485	0.471	0.218
Temp.	*rho*_CAS_	0.573	0.259	0.266	0.520	−0.357	0.441	−0.175	−0.046
	*p*_-value_	0.055	0.417	0.404	0.080	0.254	0.151	0.586	0.888
DO	*rho*_CAS_	0.140	−0.007	0.189	0.077	0.213	−0.282	0.450	−0.153
	*p*_-value_	0.667	0.991	0.558	0.817	0.505	0.374	0.142	0.636
		AGS							
		Vroomshoop				Garmerwolde			
		F-specific RNA	*Enterococci*	TtC	*E. coli*	F-specific RNA	*Enterococci*	TtC	*E. coli*
NH_4_–N	*rho*_AGS_	0.238	−0.329	−0.335	−0421	0.839	0.797	0.622	0.343
	*p*_-value_	0.457	0.297	0.287	0.173	**0.001**	**0.003**	**0.035**	0.301
BOD_5_	*rho*_AGS_	0.531	−0.070	0.153	0.245	0.615	0.650	0.713	0.636
	*p*_-value_	**0.049**	0.834	0.635	0.444	**0.037**	**0.026**	**0.012**	**0.030**
COD	*rho*_AGS_	0.329	0.392	0.573	0.426	0.643	0.685	0.685	0.503
	*p*_-value_	0.297	0.210	0.055	0.169	**0.028**	**0.017**	**0.017**	0.099
PO_4_–P	*rho*_AGS_	0.664	0.356	0.056	0.636	0.755	0.566	0.716	−0.133
	*p*_-value_	**0.002**	0.256	0.869	**0.030**	**0.006**	**0.049**	0.119	0.683
TSS	*rho*_AGS_	0.301	−0.392	−0.168	0.287	0.455	0.426	0.336	0.755
	*p*_-value_	0.343	0.210	0.604	0.366	0.140	0.169	0.287	**0.007**
Temp.	*rho*_AGS_	0.671	0.063	−0.392	0.427	−0.140	−0.312	−0.312	−0.154
	*p*_-value_	**0.020**	0.852	0.210	0.169	0.664	0.324	0.324	0.633
DO	*rho*_AGS_	0.007	−0.245	−0.007	0.119	0.385	0.455	0.615	0.098
	*p*_-value_	0.991	0.444	0.991	0.716	0.218	0.140	**0.037**	0.766

Overall, the physicochemical water quality parameters removal efficiencies strongly correlated with each other at both WWTPs (data not shown), but apparently not to all the observed FIOs removal. For the CAS process at Vroomshoop, F-specific RNA bacteriophages showed a significant correlation with most of the studied physicochemical parameters. Moreover, both the *Enterococci* and the *E. coli* removal had a significant correlation with NH_4_–N, PO_4_–P and TSS. Conversely, TtC removal showed no substantial association with any of the measured parameters. For the Garmerwolde CAS process, a significant correlation was observed between F-specific RNA bacteriophages and the chemical PO_4_–P removal and the TSS. *Enterococcus,* TtC and *E. coli* did not significantly correlate with any of the studied parameters.

For the AGS system located at Vroomshoop, the F-specific RNA bacteriophages removal showed a significant correlation with the BOD_5_, PO_4_–P removals, and it was the only indicator affected by water temperature changes (Table 3). Except for *E. coli*, which was positively correlated with the PO_4_–P, any bacteria indicator significantly correlated with the removals of any of the studied water quality parameters. For the AGS at Garmerwolde, F-specific RNA bacteriophages and *Enterococcus* were positively correlated with the chemical parameters NH_4_–N, BOD_5_, COD, and PO_4_–P, while *E. coli* showed a positive correlation with the physical parameter TSS. TtC were associated with NH_4_–N COD, BOD_5_, and it was the only indicator that showed to be significantly affected by the DO concentration.

## 4 Discussion

The main objective of this study was to compare the removal of faecal indicators in AGS systems and CAS systems. To this purpose, detection and enumeration of F-specific RNA bacteriophages, *E. coli*, *Enterococci* and TtC were analysed in raw and the treated wastewater of two full-scale WWTPs in the Netherlands.

The CAS and AGS systems studied were both engineered for domestic wastewater treatment. They manage to efficiently remove representative water quality parameters such as organic matter, TSS and total nitrogen and phosphorous, this in line with the EU Water Directive (91/271/EEC) on Urban Waste Water Treatment. CAS systems are based on interaction between bacteria and sewage with relatively large land requirements for biological treatment, sludge recycling and separation (Henze et al., 2008). The better understanding on the interaction between substrate and bacteria led the development of the AGS system in which denser granular biomass is formed and simultaneously remove organic matter and nutrients in only one biological tank (de Kreuk et al., 2005). This improvement has resulted in energy savings (20–50%) while compared to CAS systems (Pronk et al., 2017) and other technologies such as membrane bioreactors (Purnell et al., 2016). Moreover, less area is required compared to CAS systems or other technologies such as trickling filters and constructed wetlands (Stefanakis et al., 2019). Those processes normally include primary treatment and in the case of CAS a secondary sedimentation tank after the biological treatment to separate sludge from the liquid bulk. In this study, both studied WWTPs Vroomshoop and Garmerwolde lack primary settling tanks. Therefore, for the comparison of the FIOs removal, it was only needed to consider the different biological treatment and different separation processes, which is either through the settling tanks in the CAS systems or in the same tank for the AGS systems.

### 4.1 FIOs concentrations in raw and treated wastewater

The expected concentrations of F-specific RNA bacteriophages in raw wastewater has been reported to range approximately from 10^4^ to 10^6^ PFU/100 mL (De Luca et al., 2013; McMinn et al., 2017; Thwaites et al., 2018; Zhang and Farahbakhsh, 2007). The concentration of faecal coliforms (*E. coli*, TtC and *Enterococcus*) in raw wastewater ranges from 10^6^ to 10^7^ CFU/100 mL (Henze et al., 2008); Chahal et al. (2016) reported for *E. coli* concentrations of 10^4^ to 10^9^ CFU/100 mL, and Matthews et al. (2010) reported for *Enterococcus* from 10^5^ to 10^6^ CFU/100 mL. The arithmetic mean concentrations of those types of organisms in raw wastewater analysed in the current study agrees with previous studies.

### 4.2 Log_10_ removal

The Log_10_ removal of F-specific RNA bacteriophages have been reported to range from 1.5 up to 2.8 in CAS systems (Hata et al., 2013; Ulbricht et al., 2013). The results obtained in our study are in accordance with those reported values. Regarding the faecal indicator bacteria, the determined Log_10_ removal values for the CAS systems in our research at both Vroomshoop and Garmerwolde WWTPs ranged from 1.1 to 1.9, slightly lower than the values reported in the literature. The microbiological methods are standardized, therefore the differences between the reported data and the present study might be because our CAS systems are compared with CAS systems which have primary and sometimes tertiary treatment. For example, Ottoson et al. (2006a) reported removal values of 3.2 ± 0.8, 3.2 ± 0.1 and 3.5 ± 0.85 for *E. coli*, *Enterococcus* and F-specific bacteriophages in a CAS system with primary sedimentation and sand filters. Little is known about the specific contribution per unit on the removal efficiency. However, not strong contribution of FIOs removal was observed after a pre-treatment stage being an underground septic tank (Purnell et al., 2016) and a primary sedimentation tank (Stefanakis et al., 2019). Therefore, the sand filtration unit must have increased the FIOs removal rates in case of Ottoson et al. (2006a).

In this study, the only significant difference in FIOs removal measured, when comparing the CAS process at Garmerwolde WWTP with the CAS process at Vroomshoop WWTP, was for the F-specific RNA bacteriophages (2.1 Log_10_ versus 1. 3 Log_10_). However, when looking at Fig. 2, the average values for all FIOs removal were higher for Garmerwolde CAS process compared to Vroomshoop. This can be explained by reviewing the pathogen removal mechanisms in wastewater treatment systems. It may be expected that due to the FIOs isoelectric point, they get attached to the flocs and then end up the sludge line - with a high probability to remain alive in the sludge cake (Guzman et al., 2007). Due to the ferric iron dosing in the unit A, which contributes to the floc formation, the two stage AB-CAS process in Garmerwolde WWTP might enable extra FIOs removal through flocculation. Vroomshoop is operated as a carrousel and does not have ferric iron dosing. Moreover, the CAS system at Vroomshoop WWTP receives the excess/waste sludge from the AGS system (Pronk et al., 2017), which may result in an additional accumulation of FIOs in the biological tank. In Garmerwolde WWTP, the excess sludge is directly discharged to the sludge digestion line.

Another removal mechanism in CAS systems, such as biological predation in the aeration tank by protozoa organisms, have been broadly studied (Madoni, 1994,a; 1994,b). Predation might equally affect bacteriophages and faecal bacteria, but an extra biological removal mechanism might occur for bacteria such as cell lysis using *E. coli* or *Enterococcus* as preferred organism for human virus and bacteriophages replication (Young, 1992). Reproduction of the host bacteria is a prerequisite to get infected by bacteriophages (Grabow, 2001). Since the experimental period of this study was carried out during winter and spring with a low range of temperature, bacteria lysis by bacteriophages replication likely not contributed in the removal. In line with Wen et al. (2009) findings, the overall low bacterial and viral indicators removal efficiencies found in this study could have been influenced by the low temperatures in which the campaign was carried out (<15 °C). The lower the temperature, lesser amount of bacteria is expected to be adsorbed into a solid phase. Those effects can be due to changes of the water/microbial surface viscosity, reduction of some chemical/physical adsorption properties and changes physiology of the organisms (Hendricks et al., 1979; Stevik et al., 2004).

With respect to the two AGS systems (Vroomshoop and Garmerwolde WWTPs), both systems showed a similar FIOs removal efficiency. Results indicate that the differences in the flow treated and the treated organic loading rate had little impact on the FIOs removal. When comparing AGS with CAS systems, the FIOs removal in the two AGS systems showed to be statistically similar as in the CAS systems for the two WWTPs. Our results are in accordance with a previous study conducted by Thwaites et al. (2018) who compared the removal of related FIOs in pilot scale AGS and CAS systems. The design and operational conditions of the full-scale AGS systems such as absence of primary treatment, solid/liquid separation occurring in the same biological reactor, a short sedimentation period and shorter HRT than in the CAS systems may have an effect on the FIOs removal mechanisms. However, although different mechanisms might be taking place in the CAS and AGS systems, the overall amount of FIOs removal was found to be similar. The operational conditions of an AGS system determine the formation of a spherical shaped granule which surface can function as adsorption area for FIOs. Little is known about whether FIOs can be adsorbed or attached to the surface of the granular biomass. Thwaites et al. (2018), applied a method to compare the detachment of FIOs from particulate materials in CAS and mature AGS. Contrary to the F- specific RNA bacteriophages, their results showed larger separation of sulphite-reducing clostridia, *E. coli* and total coliforms from the AGS than CAS. The applied technique was not selective enough to distinguish which mechanism (absorption or single adhesion to the granule surface) was involved, thus additional studies were recommended to understand the contribution of the granular composition in the FIOs removals.

Moreover, attachment of ciliate protozoa on the surface area of the granules can also take place, facilitating biological predation of the FIOs by the protozoa (Li et al., 2013; Thwaites et al., 2018; Weber et al., 2007). The high biomass density in the AGS systems can also be a limitation for bacteriophages propagation since the granules may provide shelter to host bacteria (Rabinovitch et al., 2003). Therefore, further studies are needed to better understand the AGS process and how it relates to the pathogen removal mechanisms.

### 4.3 Correlation between microbial organisms and water quality related parameters

Correlations between bacteriophages, bacteria indicators and physicochemical parameter removal and wastewater influent concentrations reported in this study are in accordance with previous literature (De Luca et al., 2013; McMinn et al., 2017; Purnell et al., 2016). In agreement with De Luca et al. (2013), the physicochemical parameters measured in this study better correlated with each other than with the target microorganism removals. Ottoson et al., 2006a, Ottoson et al., 2006b reported removal trends between FIOs COD and total organic carbon in a CAS system. For the AGS process at Garmerwolde, correlations between the removal of the FIOs and COD, BOD_5_ and NH_4_–N were found. *E. coli* removal strongly correlated with the TSS removal in the AGS system at Garmerwolde WWTPs. According to van Dijk et al. (2018), the rapid liquid bulk separation from the biomass carried out in the biological tank of the AGS system causes a disturbance of the biomass during feeding which lead to wash-out of particles measured as TSS in the effluent, which might also include FIOs. The rest of the measured physicochemical water quality related parameters were randomly correlated with the FIOs removal for both treatment systems, and could not be used to predict FIOs removal in this study.

### 4.4 Perspective

This research showed the overall removal efficiency of two different full-scale AGS processes and compared them with the parallel CAS systems. Future priorities should be given to determine how the pathogens are removed, specifically in the AGS process. It is interesting to ascertain to what extend the short settling time or the design configuration of the AGS process affects the pathogen removal rate. The granular biofilm may also provide a large available surface area for bacteria adhesion, but may also play a significant role as carrier material for protozoa ciliates. An understanding of the dynamics in the biological tank such as microbial community, granule morphology and association with predators, might help in clarifying the pathogen removal mechanisms in the AGS systems.

## 5 Conclusions

• This work describes for the first time the removal efficiency of faecal indicators at full-scale AGS systems over a period of six months, compared with conventional activated sludge systems. Valuable information is provided for topics associated to human health risk, treated effluent discharge regulations, post-treatment constraints and disinfection matters.
• The faecal indicator (F-specific RNA bacteriophages, *E. coli*, *Enterococci*, and TtC) concentrations in the raw influent wastewater of two WWTPs in the Netherlands, Vroomshoop and Garmerwolde were found to be comparable with those reported in literature.
• It can be concluded that on average FIOs (statistically significant for F-specific RNA bacteriophages only) were removed less efficiently in a more simple CAS configuration (carrousel at the Vroomshoop WWTP) compared to a more complex one (AB-CAS system at the Garmerwolde WWTP), which might be related to the sludge separation and discharge.
• The results of this study showed that the researched AGS systems remove F-specific RNA bacteriophages, *E. coli*, *Enterococci*, and TtC as efficient as CAS systems when treating the same raw influent wastewater. However, further studies need to be done to determine in how far the removal mechanisms in both systems are comparable.
• Finally, the F-specific RNA bacteriophage better correlated with NH_4_–N, BOD_5_, COD, PO_4_–P and TSS removals than the rest of the bacteria indicators at the two evaluated WWTPs; the measured water quality parameters could not accurately predict FIOs removal at any of the evaluated treatment systems.

## Declaration of competing interest

The authors declare that they have no known competing financial interests or personal relationships that could have appeared to influence the work reported in this paper.
