# Supplementary material for: Removal of bacterial and viral indicator organisms in full-scale aerobic granular sludge and conventional activated sludge systems
Source: Water Res X. 2019 Dec 26;6:100040. doi: 10.1016/j.wroa.2019.100040 (PMC6940708; doi:10.1016/j.wroa.2019.100040)
Supplement: Multimedia component 1 [file mmc1.docx]

**Supplementary material**

**Removal of bacterial and viral indicator organisms in full-scale aerobic granular sludge and conventional activated sludge systems**

Mary Luz Barrios-Hernández ^a, b, *^, Mario Pronk ^b, c^, Hector Garcia ^a^, Arne Boersma^c^, Damir Brdjanovic ^a, b^, Mark C.M. van Loosdrecht ^b^ Christine M Hooijmans ^a^

^a^ Department of Environmental Engineering and Water Technology, IHE-Delft Institute for Water Education, P.O. Box 3015, 2601 DA Delft, The Netherlands.

^b^ Department of Biotechnology, Delft University of Technology, Van der Maasweg 9, 2629 HZ Delft, The Netherlands.

^c^ Royal HaskoningDHV B.V., P.O Box 1132,3800 BC Amersfoort, The Netherlands

Table S1. Spearmen rank correlation obtained by comparing the FIOs concentration at the influent and the physicochemical parameters concentrations per WWTP. Values stand for *rho* and *p*-value.

|  | Vroomshoop | | | |  | Garmerwolde | | | |
| --- | --- | --- | --- | --- | --- | --- | --- | --- | --- |
|  | F-specific RNA | *Enterococci* | TtC | *E. coli* |  | F-specific RNA | *Enterococci* | TtC | *E. coli* |
| *Enterococci* | -0.084 |  |  |  |  | 0.462 |  |  |  |
|  | 0.800 |  |  |  |  | 0.134 |  |  |  |
| TtC | 0.399 | 0.658 |  |  |  | 0.559 | 0.483 |  |  |
|  | 0.199 | **0.019** |  |  |  | 0.063 | 0.115 |  |  |
| *E. coli* | 0.410 | 0.326 | 0.777 |  |  | 0.582 | 0.368 | 0.067 |  |
|  | 0.186 | 0.302 | **0.003** |  |  | **0.047** | 0.239 | 0.837 |  |
| NH4-N | 0.776 | 0.119 | 0.398 | 0.427 |  | 0.860 | 0.601 | 0.611 | 0.674 |
|  | **0.005** | 0.716 | 0.240 | 0.166 |  | **0.001** | **0.043** | **0.020** | **0.016** |
| BOD_5_ | 0.846 | 0.133 | 0.350 | 0.207 |  | 0.678 | 0.608 | 0.720 | 0.611 |
|  | **0.001** | 0.683 | 0.264 | 0.519 |  | **0.018** | **0.040** | **0.011** | **0.035** |
| COD | 0.783 | 0.105 | 0.613 | 0.637 |  | 0.762 | 0.608 | 0.734 | 0.533 |
|  | **0.004** | 0.750 | 0.034 | 0.026 |  | **0.006** | **0.040** | **0.009** | 0.074 |
| PO_4_-P | 0.839 | 0.105 | 0.434 | 0.543 |  | 0.825 | 0.545 | 0.573 | 0.747 |
|  | **0.001** | 0.750 | 0.158 | 0.067 |  | **0.002** | 0.071 | 0.055 | **0.005** |
| TSS | 0.469 | 0.161 | 0.287 | 0.332 |  | 0.622 | 0.545 | 0.503 | 0.646 |
|  | 0.128 | 0.619 | 0.365 | 0.291 |  | **0.035** | 0.071 | 0.099 | **0.023** |

Table S2. Pearson products and *p*-values obtained for correlating the bacteriophages removal with bacteria indicator at Vroomshoop WWTP. Values stand for *rho* and *p*-value.

|  | Vroomshoop WWTP | | | | | |
| --- | --- | --- | --- | --- | --- | --- |
|  | CAS | | | AGS | | |
|  | F-specific RNA | *Enterococci* | TtC | F-specific RNA | *Enterococci* | TtC |
| *Enterococci* | 0.45 |  |  | 0.43 |  |  |
|  | 0.14 |  |  | 0.16 |  |  |
| TtC | 0.12 | 0.82 |  | 0.05 | 0.61 |  |
|  | 0.68 | **<<0.05** |  | 0.89 | **0.04** |  |
| *E. coli* | 0.38 | 0.82 | 0.70 | 0.46 | 0.69 | 0.54 |
|  | 0.78 | **<<0.05** | **0.01** | 0.14 | **0.01** | 0.07 |
|  | Garmerwolde WWTP | | | | | |
|  | CAS | | | AGS | | |
|  | F-specific RNA | *Enterococci* | TtC | F-specific RNA | *Enterococci* | TtC |
| *Enterococci* | 0.02 |  |  | 0.85 |  |  |
|  | 0.95 |  |  | **<<0.05** |  |  |
| TtC | 0.63 | 0.29 |  | 0.38 | 0.60 |  |
|  | **0.03** | 0.37 |  | 0.22 | **0.04** |  |
| *E. coli* | 0.45 | 0.66 | 0.53 | 0.24 | 0.35 | 0.50 |
|  | 0.14 | **0.02** | 0.85 | 0.46 | 0.27 | 0.10 |
